# Dopamine D4 receptor gene and religious affiliation correlate with dictator game altruism in males and not females: evidence for gender-sensitive gene × culture interaction

**DOI:** 10.3389/fnins.2015.00338

**Published:** 2015-09-24

**Authors:** Yushi Jiang, Rachel Bachner-Melman, Soo Hong Chew, Richard P. Ebstein

**Affiliations:** ^1^Department of Economics, National University of SingaporeSingapore, Singapore; ^2^School of Social and Community Sciences, Ruppin Academic CenterEmek Hefer, Israel; ^3^Department of Psychology, Hebrew University of JerusalemMt. Scopus, Israel; ^4^Department of Psychology, National University of SingaporeSingapore, Singapore

**Keywords:** dopamine D4 receptor, religion, altruism, gene-culture coevolution, differential susceptibility

## Abstract

On a large sample of 2288 Han Chinese undergraduates, we investigated how religion and *DRD4* are related to human altruistic giving behavior as measured with the Andreoni-Miller Dictator Game. This game enables us to clearly specify (non-)selfishness, efficiency, and fairness motives for sharing. Participants were further classified into religious categories (Christian, Buddhist-Tao, and No Religion) based on self-reports, and genotyped for the dopamine D4 receptor (*DRD4*) gene exon III VNTR. Our analysis revealed a significant interaction between religion and *DRD4* correlated with giving behavior solely among males: Whereas no significant association between religion and sharing decisions was observed in the majority 4R/4R genotype group, a significant difference in giving behavior between Christian and non-Christian males was seen in the non-4R/4R group, with Christian men being overall more altruistic (less selfish and fairer) than non-Christian men. These results support the vantage sensitivity hypothesis regarding *DRD4* that the non-4R/4R “susceptibility” genotype is more responsive to a positive environment provided by some religions.

## Introduction

“*Teach this triple truth to all: A generous heart, kind speech, and a life of service and compassion are the things which renew humanity*”

The Buddha

*Luke 14:13-14 “But when you give a reception, invite the poor, the crippled, the lame, the blind, and you will be blessed, since they do not have the means to repay you; for you will be repaid at the resurrection of the righteous*.”

Contrary to the depiction of the *Homo economicus* as purely selfish according to traditional economic theory, people are often altruistic and willing to help strangers at great cost, sacrificing even their own life. Such altruistic behaviors are usually studied in experimental economics using dictator games (Forsythe et al., 1994). Two players are randomly matched and one (“the dictator”) decides how to divide a pie between them while the other remains passive. It has consistently been shown that, contrary to the prediction of classical economic thinking, people tend to give away some of the pie, even at a cost to their own payoffs (Henrich et al., 2005).

Various evolutionary hypotheses have attempted to explain the origins of altruism, including kin selection, group selection (Eldakar and Wilson, 2011) reciprocity, reputation building (Fehr and Fischbacher, 2003; Nowak, 2006), and altruistic punishment (Fehr and Gächter, 2002). However, none of these explain human altruism completely, especially toward strangers. Cultural evolution, specifically the development of religious institutions, embodies a more egalitarian for altruism.

A unique feature of *H. sapiens* is that many skills essential for individual and group survival can be passed from one generation to the next. Such cultural evolution is apparently an important mechanism that helps explain group selection (Bell et al., 2009). Other complementary processes, such as altruistic punishment (Fehr and Fischbacher, 2003), ensure the maintenance of group social norms. Moreover, evidence supports “gene-culture coevolution,” whereby cultural and genetic forces jointly shape broad aspects of human behavior (Feldman and Laland, 1996). This theory provides a further perspective for understanding altruism (Feldman et al., 1985) and how our genome has been partly shaped by culture (Laland et al., 2010; Ross and Richerson, 2014).

Among the salient cultural factors likely important in understanding prosocial traits in humans is religion. Overall, there is considerable evidence for religious prosociality from the fields of anthropology, sociology, experimental psychology, and experimental economics (Norenzayan and Shariff, 2008; Henrich et al., 2010a). Twin studies (Koenig et al., 2007) have shown a modest correlation between religiosity and altruistic behavior. These concepts share most of their genetic influence, but only half of their shared environmental influence. Such quantitative genetic studies need to be complemented by molecular genetic approaches that inform us about specific gene contributions to the behaviors.

In Drosophila (Waddell, 2013) and across the animal kingdom (Hayes, 2013) dopamine plays a key role in human diseases such as Parkinson's and schizophrenia as well as underlying reward-driven learning in vertebrates and invertebrates. The pervasive importance of dopamine has prompted a vast literature investigating the role of dopaminergic neural transmission in both human and animal behavior. Especially following the seminal study of Schultz et al showing the importance of this molecule in reward prediction error (Schultz et al., 1997), dopamine has figured prominently in explaining the neurochemical pathways underlying altruistic and prosocial behaviors which presumably generate the “warm glow” (Kringelbach and Berridge, 2012) when people or animals engage in prosocial activity helping kin and non-kin alike. Not surprisingly then, the search for genes correlated with altruism have focused mainly on elements of the dopaminergic synapse.

A natural candidate gene that might be correlated with human altruism is the dopamine D4 receptor (*DRD4*) gene. *DRD4* is characterized by a 48 base-pair variable number tandem repeat (VNTR) in exon III, with 2–11 repeats observed in humans. In Caucasians the 4-repeat (4R) and the 7-repeat (7R) are most common (Van Tol et al., 1992). In East Asians, the 7R is virtually absent and the most common repeat after the 4R is the 2-repeat (2R; Chang et al., 1996).

The first report of an association between *DRD4* and measures of human altruism was observed in an Israeli sample using self-report questionnaire (Bachner-Melman et al., 2005), and replicated by a more recent study in a German ethnic group (Anacker et al., 2013). In both studies the absence of the exon III 7R was associated with altruism measured with self-report questionnaires. *DRD4* has also been related to reciprocal fairness measured with Ultimatum Game (Zhong et al., 2010) in Han Chinese participants, subjects homozygous for the 4R stated a 25% higher minimal acceptable offer indicating higher demand for fairness; similar finding has been reported later on among an independent Caucasian sample, where the 4/4R genotype carriers stated a 20% higher minimal acceptable offer than carriers without 4/4R genotype (mainly 7R carriers; Reuter et al., 2013). However, other studies suggest that the effect of *DRD4* on prosociality is contingent on environment (see Jiang et al., 2013 for a review). Specifically, In a study designed to evaluate environmental and genetic (G × E) influences on children's prosocial behavior Knafo et al. (2011), found that positive parenting related meaningfully to mother-rated prosocial behavior and unexplained punishment related positively to self-initiated prosocial behavior, but only among children carrying the *DRD4* 7R allele. In a Dutch study (Bakermans-Kranenburg and van Ijzendoorn, 2011), children with secure attachment representations donated money to UNICEF more but only if they had the 7R allele. Moreover, besides reporting an association between the *DRD4* exon III VNTR and reciprocal fairness, Zhong et al. (2010) also found that the interaction among this gene, season of birth, and gender was highly significant.

A first study with a very small (by standards of candidate gene investigations; Ioannidis et al., 2001, 2003) group of 178 participants was conducted by Sasaki et al. (2013). Employing a combined Asian and Caucasian sample they found a significant interaction effect of *DRD4* and religion prime on participants' prosociality, measured with their willingness to theoretically volunteer for prosocial causes supporting the environment. Specifically, people with 2R or 7R alleles were significantly more willing to volunteer when primed with religion, whereas the religion prime did not affect people without these alleles. The current investigation compared to Sasaki et al. (2013) is vigorously novel in several critical respects. First, we measure altruism in both a Western and non-Western religion in a relatively homogenous ethnic group (and not an ethnically confounded population mixture as did Sasaki et al., 2013). We underscore that the Chinese ethnic group represents 20% of the world's population and Buddhism is a religion with an estimated 350 million worldwide adherents. The current study hence is an important contribution since it goes beyond the run of the mill observations so widespread in the psychological and behavioral economic literature that generally focus on solely examining “WEIRD” people (Henrich et al., 2010b), a distinct minority in the world population. More specifically, the current study also uniquely broadens the Sasaki et al. (2013)'s finding in a subject group more than 10 fold larger than employed in the first finding therefore substantially increasing the credibility of our results. Importantly, instead of situational prime of religion, a more general context is used, and religious affiliation is assessed using a widely-used self-report questionnaire that allows a much finer grained analysis of religiosity (King et al., 1995). We also use the highly-cited Andreoni-Miller Dictator Game (Andreoni and Miller, 2002) to measure altruism that forces people to demonstrate a “put your money where your mouth is” attitude toward prosociality. This richly contoured, incentivized behavioral economic experiment identifies three well-defined types of sharing rules. Although novelty in science has many meanings, we suggest the notion that a study that establishes the credibility of an intriguing but uncertain first finding also satisfies the criteria of novelty in science viz., believability.

Participants self-identify as being Buddhist-Tao, Christian or having no religion. Both Christians and Buddhist-Taos adhere to the Golden Rule as the linchpin of human morality, albeit with different rationalizations (Reilly, 2006). In the Christian tradition we are God's children, and should therefore be treated equally, as God would treat us. In the Buddhist tradition we are suffering beings who wish to be protected from suffering just as a loving mother would protect her suffering child. Such nuanced differences in morality concepts make it interesting to compare people of these two religions and those without a religion. We hypothesized that religious affiliation would be associated with individual differences in altruism. We also hypothesized that the association between some religious groups and prosocial behavior would be more pronounced among carriers of non-4R/4R alleles. This hypothesis is based on “vantage sensitivity” (Manuck et al., 2011; Pluess and Belsky, 2013; Sweitzer et al., 2013) or “differential susceptibility” hypothesis (Ellis et al., 2011), according to which some individuals react more positively than others to the environmental advantages to which they are exposed.

## Materials and methods

### Participants

The current study is part of a larger investigation of the molecular genetic architecture of individual and other-regarding decision making. In this study, we planned to recruit 2200 undergraduate students at the National University of Singapore and we stopped recruiting with a final sample size of 2288 (1220 [53.3%] female; mean age = 20.98 year, *SD* = 1.48 year), all ethnic Han Chinese. Participants were recruited through e-mail and poster advertisements. They participated in an incentivized laboratory economic experiment session, completed online surveys including several psychological questionnaires, and provided blood samples. The study was approved by the Institution Review Board of the National University of Singapore. Participants gave informed written consent prior to participating and were reimbursed for participation in the project (S$25 per h on the average).

### Economic experiment: Andreoni-Miller (A-M) dictator game

In the original A-M DG, each subject is given a menu of choices with different endowments *m* and prices *p* for payoffs, so that π_s_ + *p*π_o_ = *m*, where π_s_ is the decision maker's own monetary payoff and π_o_ is the payoff of the other subject randomly matched with the decision maker. If Π is the set of possible payoffs of the game, a utility-maximizer *s* should choose the pair (π_s_, π_o_) ∈ Π that gives the highest level of utility of the form U_s_ = u_s_(π_s_, π_o_). Using economic models, the authors found that most observed altruistic behavior is consistent with maximization of one of the three standard CES utility functions: *perfect selfish*: u_s_(π_s_, π_o_) = π_s_, *perfect substitutes:* u_s_(π_s_, π_o_) = π_s_ + π_o_, or *Leontief* : u_s_ (π_s_, π_o_) = *min*{π_s_, π_o_}.

Our experiment involves five decision tasks (Supplementary Material) adapted from the original A-M DG. For every task, there is an initial endowment for the dictator (S$20 to S$40), and a factor *R* (picks value from set {1/3 1/2 1 2 3}) that determine the price of giving, i.e., the recipient will receive *R* dollars for every dollar sent by the dictator, while the dictator keeps the remainder. At the decision stage, each participant decides independently how much to give to the recipient in each of the five tasks. At the payment stage, participants are randomly sorted into pairs and the role of dictator and recipient are randomly assigned within each pair. One of the five decision tasks is randomly selected, and payment is made according to the dictator's decision for the task (See Supplementary Material for detailed experiment instructions).

In accordance with the utility functions defined above, we classified our participants into three types:

1. Selfish type: selfish behavior is guided by the *perfect selfish* utility. These people keep all endowments to themselves regardless of *R*;
2. Efficient type: efficiency-driven behavior is guided by the *perfect substitute* utility. These people keep all endowments when *R* is small, but give everything to the recipient when *R* is large; and
3. Fair type: egalitarian behavior is guided by *Leontief* utility. These people try to split the final payoff equally.

### Royal free interview for religious beliefs

The Royal Free Interview Questionnaire (King et al., 2001) is an instrument designed to measure religious affiliation. We asked subject which religious group they belong to and classified them into one of four categories: No Religion, Christian (including: Roman Catholic, Church of England/Anglican, Other Protestant, Evangelical Christian, and Other Christian), Buddhism and Taoism, or Other. We tried to keep the number of groups to a minimum, while preserving basic differences. Despite the differences between them, we thus decided to group various Christian denominations together, and to group Taoism together with Buddhism.

The Royal Free Interview Questionnaire also assesses the participants' spiritual beliefs via the Spiritual Scale, which sums answers to visual analog questions on the strength with which a spiritual belief is held. These questions are answered on 0–10 point Likert Scale, with high score indicating strong spiritual belief (A full list of the questions is included in Supplementary Material).

### Genotyping

Blood samples were collected from the participants, and DNA was extracted using Quiagen QIAamp DNA Blood Midi Kit. DNA was quantified with PicoGreen (Invitrogen) and DNA integrity was assessed with agarose gel electrophoresis. SNP genotyping was performed at the Genome Institute of Singapore with HumanOmniExpress12v1.0 DNA Analysis Kit (Illumina).

The exon III *DRD4* 48 bp variable number of tandem repeats polymorphism (*DRD4* exon III VNTR) was analyzed by PCR with HotStar Plus DNA polymerase and Q-solution (Qiagen). Primer sequences were: forward 5′-GCGACTACG TGGTCTACTCG-3′, reverse 5′-AGGACCC TCATGGCCTTG-3′. Thermal protocol included activation step −95°C for 15 min; 40 cycles of 94°C for 30 s, 55°C for 30 s, 72°C for 40 s; and final hold at 72°C for 5 min. PCR products were separated with electrophoresis in 1.5% agarose gel and visualized with ethidium bromide staining.

### Statistical analysis

Due to the low frequency of 7R allele in Chinese population, our analysis is conducted on the most common alleles in the sample. In particular, we combine all non-4R/4R genotypes and compare it with the 4R/4R genotype group.

To measure subjects' giving behavior, we calculate the average Giving Ratio (GR: amount sent to the recipient divided by initial endowment). Apart from the summary statistics of GR in the five tasks, we followed A-M's classification and identified three archetypical behaviors based on selfishness, efficiency and fairness motives. The deviations from the prescribed archetypical behaviors are calculated for each subject as the average absolute difference between the actual GR and predicted GR of archetypical behaviors across the five tasks. Gender differences in religious belief and sharing behavior were identified with simple *t*-tests. The correlation between *DRD4* gene and religious group as well as their interaction was examined with controlled Tobit analysis first for the whole sample and then across gender groups. The statistical analysis was performed using STATA11.

## Results

### Religious affiliation, spirituality, and DRD4 genotypes

In our sample of 2288 participants, almost half (46.2%) reported they had “no religious affiliation,” 28.6% report themselves to be Christians and 24.6% Buddhists or Taoists (B-T). The 14 participants who reported a less common religious affiliation (at least in Singapore) were excluded from subsequent analyses due to the small sample size. Among them, 1968 participants have answered all six questions regarding spiritually, with a mean Spiritual Scale of 30.275, *SD* = 15.963. Moreover, the Spiritual Scale is the highest in the Christian group (mean = 44.369, *SD* = 12.550), followed by the B-T group (mean = 28.043, *SD* = 11.041), and is the lowest in the No Religion group (mean = 20.166, *SD* = 12.657), linear regressions show significant differences in the mean values of Spiritual Scale between the three religious categories (*p* < 0.001 for all pair-wise comparisons).

Consistent with the vast findings in the literature that women tend to be more religious than men (e.g., De Vaus and McAllister, 1987), using two sided *t*-tests we find that significantly more males (49.2%) than females (42.9%) are non-religious (*t* = 2.962, *p* = 0.003), while more females (30.7%) than males (26.9%) are Christians (*t* = −1.978, *p* = 0.048); the difference is not significant for B-T (*t* = −1.277, *p* = 0.202) or Other religions (*t* = −0.347, *p* = 0.729). In the two-sided *t*-test of Spiritual Scales, we find that females (mean = 31.303, *SD* = 15.546) are significantly more spiritual than males (mean = 28.888, *SD* = 16.302) and, with *p* < 0.001.

The *DRD4* exon III VNTR was successfully genotyped in 2191 participants. The 4R was the most common allele (75.58%), followed by the 2R allele (21.07%), and the other alleles were very uncommon. When looking at the distribution of genotypes, 58.06% showed the 4R/4R genotype, with all others showing at least one minor allele. This is a similar distribution to that observed in other Eastern Asian populations (China, Korea, and Japan). There is no significant gender difference in the distribution of genotypes (*t* = 0.949, *p* = 0.343).

We also examined the distribution of religious affiliations stratified by the *DRD4* exon III VNTR genotypes. For both genotypes, around 46% (4R/4R: 45.60%, Non-4R/4R: 45.70%) subjects self-identified as No Religion, less than one third belong to the Christian group (4R/4R: 28.93%, Non-4R/4R: 28.84), and less than one quarter are in the B-T group (4R/4R: 24.92%, Non-4R/4R: 24.70%). Kolmogorov-Smirnov tests revealed no difference in religious affiliation between the 4R/4R and non-4R/4R groups (*D* = 0.002, *p* = 1.000), implying no self-identification of *DRD4* exon III repeat number with one of the three religious groups (Christian, B-T or No Religion) among our participants. Finally, a two-sided *t*-test does not find significant difference in Spiritual Scale between 4R/4R (mean = 30.371, *SD* = 15.884) and non-4R/4R (mean = 30.122, *SD* = 16.020) genotypes (*p* = 0.734). These results indicate no evidence for gene × environment correlation (rGE; Rutter et al., 2006).

### A-M DG and sharing rules

Table 1 shows the predicted GR of each archetypical behavior. As explained in Material and Methods Section, the GR is zero regardless of *R* for the Selfish type; it is zero when *R* < 1 and increases to one when *R* > 1 for the Efficient type; finally, for the Fair type, the GR guarantees the final payoff between the two players are exactly the same.

**Table 1:** **Predicted GR for the three dimensions**.

	***R* = 1/3**	***R* = 1/2**	***R* = 1**	***R* = 2**	***R* = 3**
Selfish	0	0	0	0	0
Efficient	0	0	[0, 1]	1	1
Fair	3/4	2/3	1/2	1/3	1/4

In our experiment, the observed actual average GR in the five decision-tasks vary from 0.19 to over 0.33, and increase as *R* becomes larger, implying that not all participants are selfish, and people do consider overall efficiency when making decisions. Following the A-M DG classification, we calculated for each subject how much their actual decisions deviated from the predicted archetypical behaviors shown in Table 1. Specifically, in each decision task, we calculated the absolute difference between actual GR and predicted GR for each of the three archetypes. The average difference across the five tasks, ranging from 0 to 1, was then used to measure the participant's deviation from the specific type of allocation (see Material and Methods Section for calculation details).

Overall, 458 (21.1%) of our subjects follow “pure” Selfish behavior, i.e., never share anything with others. In comparison, fewer people are characterized by the archetypical Efficient (*N* = 172; 7.9%) or pure Fair behaviors (*N* = 64; 2.9%).

Importantly, for most of our participants, their sharing decision is shaped by more than one of these three archetypes. Further scrutiny of the cumulative distributions of the deviations shows that most participants make decisions following Selfish (93.4%) and Fair (88.0%) considerations by deviating less than half-way from the archetypical behaviors (Deviation < 0.5), whereas fewer participants (71.1%) do so for the Efficient behavior. The mean value of Efficient deviation is 0.435 (*SD* = 0.190), significantly higher than the mean of Selfish (mean = 0.257 *SD* = 0.193; *t* = 30.678, *p* < 0.001) and Fair deviation (mean = 0.351 *SD* = 0.181; *t* = 15.090, *p* < 0.001) using two-sided *t*-tests. Altogether, these results indicate that self-interest and fairness were the major concerns for most of our participants when deciding how much to share, whereas efficiency does not appear to play a major role in people's decision making.

### The correlation between genotype, religion, and sharing decisions

**Figure 1:**
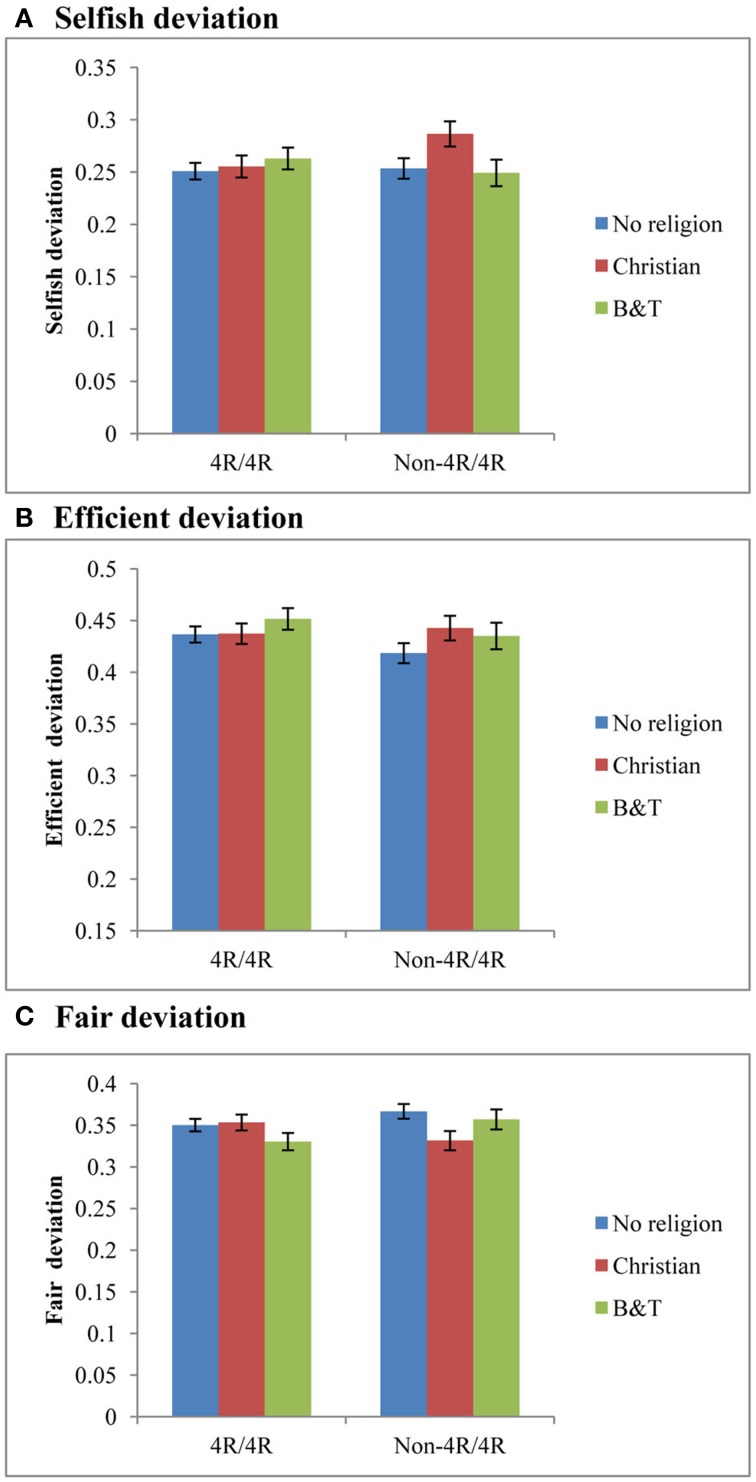
**Interaction effect of ***DRD4*** and religious affiliation**. Mean comparison of deviations from archetypical behaviors between religious affiliations, stratified by *DRD4* exon III genotype. **(A)** Shows Selfish deviation, **(B)** shows Efficient deviation, and **(C)** shows Fair deviation. The blue bars are No religion group, red bars are the Christian group, and green bars are the B-T group. Error bars are SEM.

Next, we analyze how the *DRD4* exon III genotype interacts with religion and correlates with giving decision. We firstly focus on religious affiliation. Figure 1 shows the mean comparison of deviations from the archetypical behaviors within genotype groups. Among all non-4R/4R carriers, the Christian group was least selfish, least efficient and most egalitarian. To see whether these differences are significant, we use two-sided *t*-tests to compare the mean deviations between the Christian group and non-Christians (a collapse of the B-T and the No Religion group). The results show that the mean values of the Christian group were significantly higher than non-Christian groups for Selfish deviation (*t* = 2.399; *p* = 0.017) and lower for Fair deviation (*t* = −2.373; *p* = 0.018). No significant difference was observed for the Efficient deviation (*t* = 1.288; *p* = 0.198). In contrast, there is no significant difference between the religious groups within the 4R/4R group (*p* > 0.100). Secondly, we check whether spirituality is related to giving behavior in either of the two genotype groups. Using pearson's correlation tests, we do not observe any significant correlation between the Spiritual Scale and the three deviations in either genotype group (4R/4R: Selfish deviation: *r* = 0.050, *p* = 0.101; Fair deviation: *r* = −0.008, *p* = 0.790; Efficient deviation: *r* = −0.016, *p* = 0.604; non-4R/4R: Selfish deviation: *r* = 0.004, *p* = 0.912; Fair deviation: *r* = −0.021, *p* = 0.548; Efficient deviation: *r* = 0.034, *p* = 0.340).

These preliminary findings imply that religious affiliations, but not spirituality, interact with the *DRD4* exon III genotype and correlate with giving behavior. We thus conducted controlled Tobit regressions on the three types of behavior to gain a deeper understanding on the interaction effects. In the regression we used deviations from each archetypical behavior as the dependent variable and examined their correlation with being Christian, Buddhists/Tao, carrying non-4R/4R genotype, as well as their interactions. The results are reported in Table 2, columns (1)–(3) are models without any control variable, while columns (4)–(5) are models controlling for gender and age. Before including control variables in the regression, we observe no significant main effect of religious affiliation or *DRD4* gene, but we do observe a significant interaction of being Christian with non-4R/4R genotype for Fair behavior (coeff. = −0.040, *p* = 0.041). This result suggests that Christians behave differently when carrying non-4R/4R genotypes compared with 4R/4R genotype. Put another way, the non-4R/4R genotype carriers appear to be more sensitive to the prosocial signals provided by adhering to the Christian faith. After controlling for age and gender, the Christian × Non-4R/4R interaction remains marginally significant (coeff. = −0.036, *p* = 0.068).

**Table 2:** **Tobit regression results for religion × *DRD4* Interaction**.

	**(1)**	**(2)**	**(3)**	**(4)**	**(5)**	**(6)**
	**Selfish 1**	**Efficient 1**	**Fair 1**	**Selfish 2**	**Efficient 2**	**Fair 2**
Christian	0.005	0.000	0.004	0.007	−0.002	0.004
	(0.017)	(0.014)	(0.012)	(0.017)	(0.014)	(0.013)
B-T	0.017	0.016	−0.020	0.018	0.009	−0.016
	(0.016)	(0.014)	(0.013)	(0.017)	(0.014)	(0.013)
Non-4R/4R	0.003	−0.021	0.017	0.006	−0.022	0.016
	(0.016)	(0.014)	(0.012)	(0.016)	(0.014)	(0.012)
Christian ^*^	0.036	0.027	−0.040^**^	0.030	0.027	−0.036^*^
Non-4R/4R	(0.025)	(0.022)	(0.019)	(0.025)	(0.022)	(0.020)
B-T ^*^	−0.021	0.004	0.011	−0.026	0.014	0.008
Non-4R/4R	(0.026)	(0.022)	(0.020)	(0.026)	(0.023)	(0.020)
Female				0.008	0.055^***^	−0.040^***^
				(0.012)	(0.010)	(0.009)
Age				−0.002	−0.003	0.005^*^
				(0.004)	(0.003)	(0.003)
Constant	0.223^***^	0.431^***^	0.348^***^	0.265^***^	0.469^***^	0.269^***^
	(0.010)	(0.008)	(0.008)	(0.083)	(0.072)	(0.064)
Log likelihood	−432.139	95.276	464.594	−415.236	95.862	475.961
Left censored	447	167	64	431	167	60
Observations	2138	2138	2138	2073	2073	2073

Dependent variables are deviations from prototype behaviors, and are left censored. The coefficients are presented in marginal effects. Standard errors are in parentheses.

* *p < 0.10*,

** *p < 0.05*,

*** *p < 0.01. Christian = 1 if subject belongs to the Christian group, 0 if otherwise. B-T = 1 if subject belongs to the B-T group, 0 if otherwise. Non-4R/4R = 1 if subject carries the Non-4R/4R genotype, 0 if 4R/4R genotype*.

Moreover, there is a highly significant gender difference for both Efficient and Fair behaviors: on average, females deviates 0.055 (*p* < 0.001) more from the archetypical Efficient behavior while 0.040 less from the archetypical Fair behavior (*p* < 0.001) than males, and thus are less efficiency oriented but more fair minded. Importantly, this observation confirms the notion put forward by Andreoni and Vesterlund (2001): men tend to be more responsive to price changes when making sharing decisions while women are more egalitarian, suggesting us to analyze male and female groups separately.

### Gender differences in religion × DRD4 interaction

To understand how religion and *DRD4* interact in each gender, we preformed Tobit regressions separately for both men and women. Table 3 shows that the interaction effects are only observed in men: there is a significant interaction between Christian and *DRD4* genotype, for both Selfish (coeff. = 0.087, *p* = 0.029) and Fair (coeff. = −0.081, *p* = 0.008) behavior, while no significant interactions in the Female group. This may be understood given the behavioral differences in fairness between men and women as reported in the literature (Andreoni and Vesterlund, 2001).

**Table 3:** ***DRD4* × religion interaction in male and female subgroups**.

	**Male**			**Female**		
	**(1.A)**	**(1.B)**	**(1.C)**	**(2.A)**	**(2.B)**	**(2.C)**
	**Selfish**	**Efficient**	**Fair**	**Selfish**	**Efficient**	**Fair**
Christian	−0.022	0.001	0.016	0.031	−0.005	−0.006
	(0.026)	(0.024)	(0.019)	(0.022)	(0.016)	(0.017)
B-T	−0.012	0.027	−0.017	0.042^**^	−0.004	−0.016
	(0.027)	(0.022)	(0.020)	(0.021)	(0.018)	(0.018)
Non-4R/4R	−0.009	−0.030	0.029^*^	0.022	−0.013	0.000
	(0.024)	(0.022)	(0.017)	(0.022)	(0.017)	(0.017)
Christian ^*^	0.087^**^	0.054	−0.081^***^	−0.015	0.005	0.001
Non-4R/4R	(0.040)	(0.037)	(0.030)	(0.033)	(0.026)	(0.026)
B-T ^*^	−0.001	0.012	−0.000	−0.044	0.013	0.017
Non-4R/4R	(0.041)	(0.034)	(0.029)	(0.034)	(0.029)	(0.028)
Age	−0.000	−0.000	0.001	−0.003	−0.005	0.007^*^
	(0.006)	(0.006)	(0.005)	(0.005)	(0.004)	(0.004)
Constant	0.230	0.400^***^	0.340^***^	0.286^***^	0.574^***^	0.179^**^
	(0.142)	(0.131)	(0.105)	(0.096)	(0.079)	(0.078)
Log likelihood	−261.661	−83.170	199.307	−146.094	199.634	280.606
Left censored	232	110	30	199	57	30
Observations	971	971	971	1102	1102	1102

Dependent variables are deviations from prototype behaviors, and are left censored. The coefficients are presented in marginal effects. Standard errors are in parentheses.

* *p < 0.10*,

** *p < 0.05*,

*** *p < 0.01. Christian = 1 if subject belongs to the Christian group, 0 if otherwise. B-T = 1 if subject belongs to the B-T group, 0 if otherwise. Non-4R/4R = 1 if subject carries the Non-4R/4R genotype, 0 if 4R/4R genotype*.

We next focus on the Male group to further investigate how the association between religious belief and sharing decision depends on *DRD4* genotype. We spilt the Male group into 4R/4R vs. non-4R/4R carrier groups. Similar as Figure 1; Figure S1 illustrates the average deviations from the archetypical behaviors within genotype groups, but only in Male group. As expected, no significant difference has been observed in the 4R/4R group. In comparison, when carrying non-4R/4R genotype, Christian males are significantly less selfish (*t* = −2.479, *p* = 0.014) and fairer (*t* = 2.797, *p* = 0.005) compared with non-Christian males.

Among males, we also conducted Tobit regressions to examine the effects of religious belief. Results in Table 4 show that only when carrying non-4R/4R genotypes, Christian males (*N* = 107) are significantly different from non-religious males (*N* = 206), by behaving less selfish (coeff. = 0.065, *p* = 0.029) and less efficient (coeff. = 0.055, *p* = 0.053), so as to pursue the goal of higher fairness (coeff. = −0.065, *p* = 0.006). Self-reported identification with Buddhism/Tao does not make a significant behavioral difference in our male subjects.

**Table 4:** **Effects of religion split by genotype in males**.

	**4R/4R**			**Non-4R/4R**		
	**(1.A)**	**(1.B)**	**(1.C)**	**(2.A)**	**(2.B)**	**(2.C)**
	**Selfish**	**Efficient**	**Fair**	**Selfish**	**Efficient**	**Fair**
Christian	−0.022	0.001	0.016	0.065^**^	0.055^*^	−0.065^***^
	(0.026)	(0.024)	(0.019)	(0.030)	(0.028)	(0.023)
B-T	−0.012	0.027	−0.017	−0.014	0.039	−0.016
	(0.027)	(0.022)	(0.020)	(0.031)	(0.027)	(0.022)
Age	0.003	0.000	−0.001	−0.004	−0.001	0.004
	(0.008)	(0.008)	(0.006)	(0.010)	(0.009)	(0.007)
Constant	0.162	0.383^**^	0.385^***^	0.299	0.389^*^	0.317^**^
	(0.182)	(0.172)	(0.142)	(0.220)	(0.200)	(0.157)
Log likelihood	−150.514	−34.224	106.795	−111.004	−48.413	92.632
Left censored	135	60	20	97	50	10
Observations	549	549	549	422	422	422

Dependent variables are deviations from prototype behaviors, and are left censored. The coefficients are presented in marginal effects. Standard errors are in parentheses.

* *p < 0.10*,

** *p < 0.05*,

*** *p < 0.01. Christian = 1 if subject belongs to the Christian group, 0 if otherwise. B-T = 1 if subject belongs to the B-T group, 0 if otherwise. Non-4R/4R = 1 if subject carries the Non-4R/4R genotype, 0 if 4R/4R genotype*.

## Discussion

Previously Sasaki et al. (2013) reported an interaction between *DRD4* and religion to impact prosocial behavior measured with hypothetical volunteering questions, where they found that subjects with 2R or 7R alleles are more responsive to the religion prime than subjects without these alleles. In this study, a robust incentivized behavioral economic paradigm was employed to extend these findings by uniquely examining altruism and its relationship to cultural milieu indexed by religious affiliation by both comparing two world religions as well as employing a much larger and hence more credible homogenous ethnic group. Our Tobit analysis showed the importance of *DRD4* genotype, Christianity and their interaction in sharing motives (non-selfishness/fairness/efficiency): Christianity was related to higher fairness and lower selfishness, particularly among male non-4R/4R carriers, who were not responsive to prosocial religious norms.

We conjecture that the reason only males but not females show this interaction effect is due to the overall difference between the sexes in both religious belief and prosociality. On the one hand, we observe a higher proportion of female holding Christian belief while more males are non-religious, as well as a higher Spiritual Scale among females than in males, implying higher religiosity among females; on the other hand, females are on average more concerned about fairness but less about efficiency. It is therefore plausible that for men there is more space for genetic differences in prosociality especially in the Christian milieu to be observed, while for women, who are already highly religious and prosocial, the marginal difference of the genotypes has diminished to a trivial level and thus is difficult to be detected.

It is important to note that the brain dopaminergic system is exquisitely sensitive to both male and female sex hormones. Androgen and estrogen receptors are widely distributed in the brain mesolimbic system involved in reward and motivation. Testosterone plays a critical role in the regulation of mesolimbic dopamine receptors (see review by Sotomayor-Zarate et al., 2014). Interestingly, androgens appear to have a suppressing effect on brain dopaminergic reward systems especially social reward (Bell and Sisk, 2013). We have recently shown a gender specific effect of the *DRD4* repeat polymorphism on cognitive empathy (Uzefovsky et al., 2014) suggesting the notion perhaps that gender effects × *DRD4* may indeed be the rule and not the exception. In addition to their acute effects on brain dopaminergic activity in adult animals, sex hormones have a profound effect on the developing brain inducing sexual differentiation in the brain (Arnold and Breedlove, 1985). For these reasons just discussed and previously, it is not surprising that the current findings observe an interaction between altruism and the *DRD4* receptor which is gender specific. In the clinical literature the role of *DRD4* 7R allele has been well established as an etiological factor in attention deficit hyperactivity disorder (Hawi et al., 2015), an illness much more common in males than females and these robust findings lend further plausibility to the current observation that the role of the non-4R/4R repeats in Christians with respect to altruistic giving is observed only in males.

This relationship between *DRD4* genotype and Christian milieu are consistent with the viewpoint of Belsky and colleagues (Belsky et al., 2007, 2009; Pluess and Belsky, 2013). They see *DRD4* exon III as a plasticity gene allowing behavioral fine tuning to environmental signals, with supportive and adverse contexts promoting, respectively, positive and negative outcomes. Our results provide evidence for half of this hypothesis, the “vantage sensitivity” (Manuck et al., 2011; Pluess and Belsky, 2013; Sweitzer et al., 2013). Specifically, we suggest the notion that non-4R/4R repeats confer an advantage by having its carriers being more prosocial when energized by the positive spirit of Christianity and its tradition of charity and tithing especially in the Singapore mega churches (Yip and Ainsworth, 2013). In other words, the non-4R/4R genotypes were more responsive to Christian norms promoting fairness and discouraging selfishness. Our results thus extend the concept of plasticity and sensitivity to include cultural influences at an institutional level.

Little is known about the acquisition of cultural norms like generosity and fairness, and how genes link to it. Culture may be a major environmental factor for Gene × Environmental interactions (Kim et al., 2010a,b). A specific role of the *DRD4* exon III in the acquisition of cultural norms was demonstrated by Kitayama et al. (2014). The current study shows that a major cultural institution, Christianity, interacts with this repeat region that correlates with human altruism. Non-4R/4R repeats were shown to be a sensitive tuning fork to environmental signals that can resonate with the religious cultural milieu.

Notably, in our study *DRD4* is not related with altruism in the Buddhist/Taoist group, although they, like Christianity, emphasize charitable giving. This may be in part because Taoism and Buddhism are observed differently from day to day. Additionally, the religious profile of the Singapore student population (25% Buddist/Tao, 29% Christian ~45% no religion) differs from that of the general population (44% Buddist/Tao, 18.3% Christian 17% no religion; Statistics, 2011). The younger generation of educated Chinese seems to be disavowing traditional religion (Buddhist-Taoist) for Christianity. Interestingly, converts stated that Christianity had a distinct, comprehensible set of texts and organized structure, with regular services, “Sunday school,” Bible study, notably tithing (Goh, 2011) and fellowship. Buddhist and Tao religious observance involves relatively infrequence attendance of religious services (Buddhanet, 2008), suggesting that Buddhism and Taoism “market” religion less effectively than the Christian churches in Singapore (Yip and Ainsworth, 2013) and the growth of mega-churches in Singapore is especially noteworthy (Chong and Hui, 2013). Hence, we suggest that the differences we observed between the Christians and B-T group is understandable despite apparently similar theological and theoretical attitudes toward charity. As shown in our analysis, Christians have much higher spiritual scales than Buddhists and Taoists, although spirituality does not directly contribute to prosocial behavior, a more spiritual person may be more willing to be exposed to religious environments and to accept its teaching. We suggest that the evidence points to Christianity in Singapore perhaps being a stronger factor in peoples' behavior than the more traditional Buddhism/Taoism. We note that the reasons for this difference as we have suggested, however, are somewhat speculative.

Our results show that by enhancing altruism, some religions(Norenzayan and Shariff, 2008) becomes a force in group survival, and moreover, that religion may drive human evolution by selecting for genes that link to altruistic choices. The *DRD4* gene is extremely polymorphic and stands out as a plausible candidate in this regard. By implementing a molecular genetic strategy we show that religion is related with altruistic giving behavior, but only in a specific group of Christian participants characterized by a certain genotype. Hence a molecular genetic approach is valuable in understanding human psychological and economic preferences (Ebstein et al., 2010).

Since 4R and 2R are the most prevalent alleles in our sample (96.7% in total), to maximize the usage of genetic information, we classify our participants into 4R/4R genotype and non-4R/4R genotypes. For Caucasians samples, such classification yields similar results as comparing 4R/4R with 7R carriers. For East Asian samples, this would be similar to 4R/4R vs. 2R comparison, as commonly employed in East Asian participants (Kang et al., 2010; Sasaki et al., 2013; Lim et al., 2012). Recent molecular studies of DRD4 receptor synthesis (Van Craenenbroeck et al., 2005, 2011) show that the folding efficiency is rate-limiting in the biogenesis of 4R, and the 2R is less up-regulated than the 4R. Additionally, our grouping of 4R/4R vs. all other genotypes assumes that these minor alleles are less efficient alleles derived from the more ancestral 4R, as perhaps suggested by their lower frequency (Tovo-Rodrigues et al., 2012).

A main strength of this study is a strong biologically plausible hypothesis for the joint role of *DRD4* and religiousness in relation to altruism, based on previous studies (Bachner-Melman et al., 2005; Zhong et al., 2010; Bakermans-Kranenburg and van Ijzendoorn, 2011; Knafo et al., 2011; Sasaki et al., 2013; Anacker et al., 2013). Additionally, to minimize the problem of population stratification we recruited university students from a single well-characterized ethnic group, Singaporean Han Chinese. To our knowledge this is also the largest study of Gene × Cultural interaction for this particular gene and one of the largest in behavioral genetics. It not only confirms previous finding of the between religion, *DRD4* gene and altruistic behavior in an independent large sample, but also extends it by a more generalized context and well-defined experimental measures in a distinct ethnic group. Of course the best insurance against a false positive result is replication and we expect the current report will catalyze future large-scale studies toward unraveling the role of specific genes, religion and their joint role in correlation with human altruism.

## Author contributions

RE developed the study concept. All authors contributed to the study design. YJ performed testing, data collection, data analysis, and interpretation under the supervision of RE and SHC. YJ and RB drafted the manuscript, and RE and SHC provided critical revisions. All authors approved the final version of the manuscript for submission.

### Conflict of interest statement

The authors declare that the research was conducted in the absence of any commercial or financial relationships that could be construed as a potential conflict of interest.

## Supplementary material

Figure S1 **Interaction effect of *DRD4* and religious affiliation in Male subgroup**. Mean comparison of deviations from archetypical behaviors between religious affiliations, stratified by *DRD4* exon III genotype (only using data from males and not females, as similar pattern is not observed in the Female subgroup). **(A)** Shows Selfish deviation, **(B)** shows Efficient deviation, and **(C)** shows Fair deviation. The blue bars are No religion group, red bars are the Christian group, and green bars are the B-T group. Error bars are SEM.

## Abbreviations

*DRD4*: dopamine D4 receptor
VNTR: variable number of tandem repeats polymorphism
4R: 4-Repeat
7R: 7-Repeat
2R: 2-Repeat
A-M DG: Andreoni-Miller Dictator Game
B-T: Buddhists or Taoists
GR: Giving Ratios.
