# Supplementary figures and images for: Dopamine D4 receptor gene and religious affiliation correlate with dictator game altruism in males and not females: evidence for gender-sensitive gene × culture interaction
Source: Front Neurosci. 2015 Sep 24;9:338. doi: 10.3389/fnins.2015.00338 (PMC4585304; doi:10.3389/fnins.2015.00338)

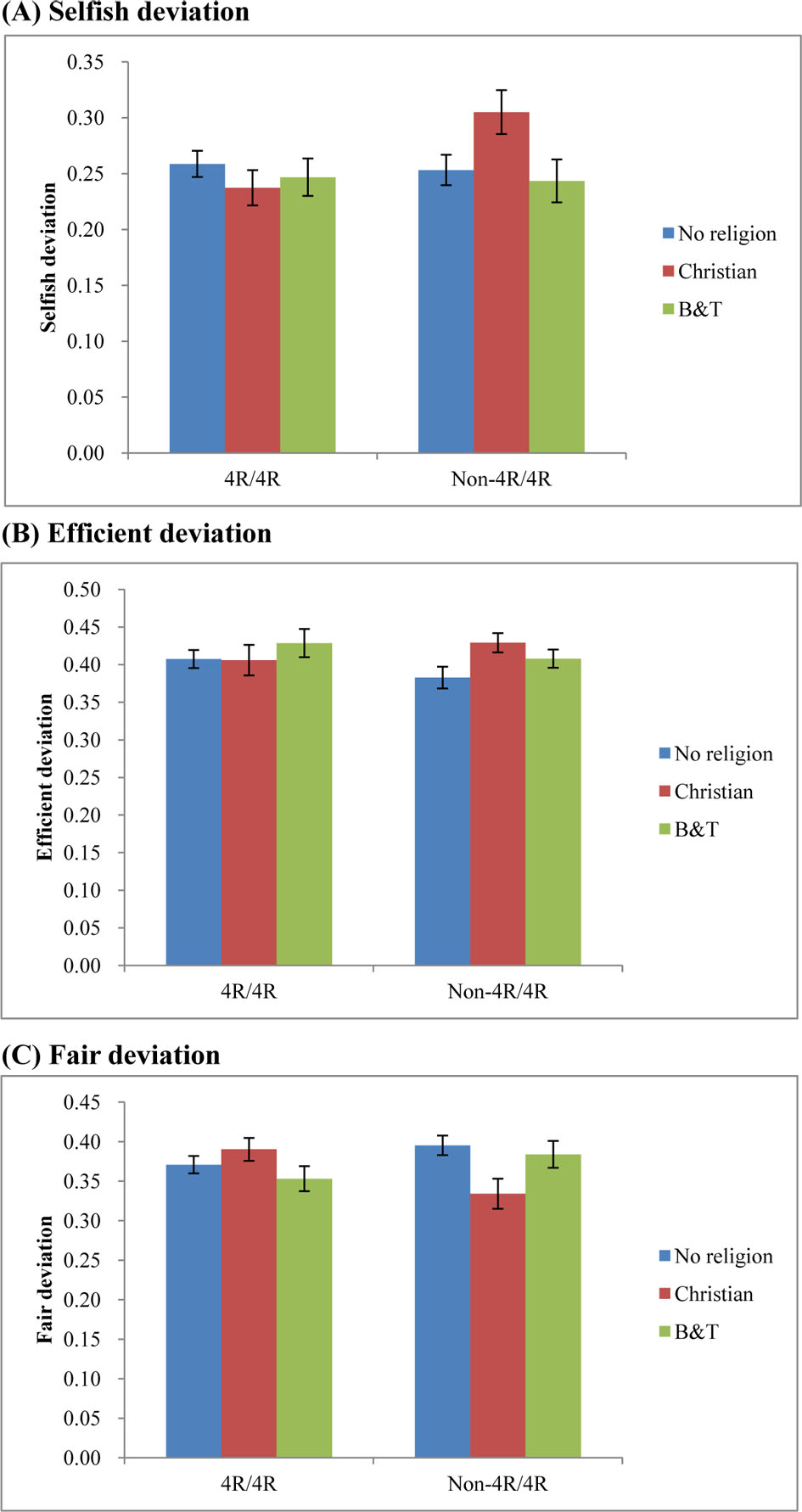

Supplement: Figure S1 — Interaction effect of DRD4 and religious affiliation in Male subgroup. Mean comparison of deviations from archetypical behaviors between religious affiliations, stratified by DRD4 exon III genotype (only using data from males and not females, as similar pattern is not observed in the Female subgroup). (A) Shows Selfish deviation, (B) shows Efficient deviation, and (C) shows Fair deviation. The blue bars are No religion group, red bars are the Christian group, and green bars are the B-T group. Error bars are SEM. [file FigureS1.JPEG]
