# Supplementary material for: Dopamine D4 receptor gene and religious affiliation correlate with dictator game altruism in males and not females: evidence for gender-sensitive gene × culture interaction
Source: Front Neurosci. 2015 Sep 24;9:338. doi: 10.3389/fnins.2015.00338 (PMC4585304; doi:10.3389/fnins.2015.00338)
Supplement: Supplementary file 2 [file Text1.PDF]

**Supplemental Material**

1. Experimental Instruction

In this situation, **Person A** is endowed a fixed amount of money, and is asked what amount of money he/she wants to send to **Person B**. Person B makes no decision. The amount of money Person A sends to Person B will be multiplied by a **factor R.** That is, Person B will receive R dollars for every dollar sent by Person A. The amounts that Person A and Person B receive depend solely on how Person A decides to allocate the money.

Example 1 (Endowed with **$50**; factor **R = 2**): *Person A can either keep all $50, keep some and send the balance, or send all of $50 to an anonymous and randomly matched Person B. For every dollar sent by Person A, Person B will receive $2. If Person A keeps $50 and sends $0, Person B will receive $0 while Person A will keep $50. If Person A sends all $50, Person B will receive $50 x 2 = $100 while Person A will have $0.*

Example 2: (Endowed with **$30**; factor **R = 1/3**)*: Person A can send up to $30. Person B receives $1/3 for every dollar sent by Person A.*

**Your decision as Person A: For each of the 5 cases** below, please indicate the amount you would keep and the amount you would send. In each case, the sum of the amount of money you keep and the amount to be sent must **equal** your endowed amount as shown in the final column.

| | **Endowed Amount** | **Factor R** | **Amount Kept** | **Amount Sent** | **Total** |
|---|---|---|---|---|---|
| 1 | $40 | 2 | | | $40 |
| 2 | $20 | 3 | | | $20 |
| 3 | $40 | 1/2 | | | $40 |
| 4 | $60 | 1/3 | | | $60 |
| 5 | $30 | 1 | | | $30 |

2. Spirituality Questions

We are now going to ask you some questions about religious and spiritual beliefs. Please try to answer them even if you have little interest in religion.

In using the word religion, we mean the actual practice of a faith, e.g. going to a temple, mosque, church or synagogue. Some people do not follow a specific religion but do have spiritual beliefs or experiences. For example, they may believe that there is some power or force other than themselves that might influence their life. Some people think of this as God or gods, others do not. Some people make sense of their lives without any religious or spiritual belief.

1. Therefore, would you say that you have a religious or spiritual understanding of your life? (Please tick one or more.)

Religious □ Religious and spiritual □ Spiritual □ Neither religious nor spiritual

2. Some people hold strongly to their views and others do not. How strongly do you hold to your religious/spiritual view of life? Circle the number that best describes your view.

Weakly 01 2 3 4 5 6 7 8 9 10 Strongly held view held view

3. Do you have a specific religion?

I do not observe a religion □Roman Catholic □ Church of England'Anglican □ Other Protestant □ Evangelical Christian □ Other Christian □ Shi'ite Moslem □ Sunni Moslem □ Jew □ Hindu □ Jain □Sikh □ Buddhist □ Others

4. How important to you is the practice of your belief (e.g. private meditation, religious services) in your day- to-day life? Please circle the number on the scale which best describes your view.
Not 0 1 2 3 4 5 6 7 8 9 10 Essential necessary

5. Do you believe in a spiritual power or force other than yourself that can

influence what happens to you in our day-to-day life? Please circle the number on the scale which best describes your view.
No 0 1 2 3 4 5 6 7 8 9 10 Strong influence influence

6. Do you believe in a spiritual power or force other than yourself that enables you to cope personally with events in your life? Please circle the number on the scale which best describes your view.
No 0 1 2 3 4 5 6 7 8 9 10 A great help

7. Do you believe in a spiritual power or force other than yourself that influences world affairs, e.g. wars? Please circle the number on the scale which best describes your view.
No 0 1 2 3 4 5 6 7 8 9 10 Strong influence

8. Do you believe in a spiritual power or force other than yourself that influences natural disasters, like earthquakes, floods? Please circle the number on the scale which best describes your view.

No 0 1 2 3 4 5 6 7 8 9 10 Strong influence
